# Supplementary material for: Rough set based information theoretic approach for clustering uncertain categorical data
Source: PLoS One. 2022 May 13;17(5):e0265190. doi: 10.1371/journal.pone.0265190 (PMC9106167; doi:10.1371/journal.pone.0265190)
Supplement: Attachment — Submitted filename: Response to Reviewers.docx [file pone.0265190.s001.docx]

***ACADEMIC EDITOR:***

When submitting the revised paper, please also consider the following points: ***Comment: 1. English language needs proofreading.***

**Response:** English proof reading is done and as per capability all relevant issues are resolved.

***Comment: 2. References should be in proper format.***

**Response:** References are reviewed to remove any mistake.

***Comment: 3. All acronyms must first be defined.***

**Response:** Acronyms are defined at first place.

***==============================
JOURNAL REQUIREMENTS:***

When submitting your revision, we need you to address these additional requirements.

***Comment: 1. Please ensure that your manuscript meets PLOS ONE's style requirements, including those for file naming. The PLOS ONE style templates can be found at*** 
[***https://journals.plos.org/plosone/s/file?id=wjVg/PLOSOne_formatting_sample_main_body.pdf***](https://journals.plos.org/plosone/s/file?id=wjVg/PLOSOne_formatting_sample_main_body.pdf) ***and*** 
[***https://journals.plos.org/plosone/s/file?id=ba62/PLOSOne_formatting_sample_title_authors_affiliations.pdf***](https://journals.plos.org/plosone/s/file?id=ba62/PLOSOne_formatting_sample_title_authors_affiliations.pdf)***.***

**Response:** All above requirements are considered in preparing the revised manuscript.

***2. Thank you for stating the following in the Acknowledgments Section of your manuscript: 
[The authors would like to thank the King Khalid University of Saudi Arabia for 
supporting this research under grant number R.G.P.1/365/42.]***

***Comment: 2. We note that you have provided funding information that is currently declared in your Funding Statement. However, funding information should not appear in the Acknowledgments section or other areas of your manuscript. We will only publish funding information present in the Funding Statement section of the online submission form. 
Please remove any funding-related text from the manuscript and let us know how you would like to update your Funding Statement.***

***Currently, your Funding Statement reads as follows: 
 [The authors would like to thank the King Khalid University of Saudi Arabia for supporting this research under grant number R.G.P.1/365/42.]***

***Please include your amended statements within your cover letter; we will change the online submission form on your behalf.***

**Response:** The funding-related text is now removed from the manuscript.

***Comment: 3. Please include a separate caption for each figure in your manuscript.***

**Response:** The caption of Figures is now included as per instructions.

***==============================***

***REVIEWERS' COMMENTS:
Reviewer #1: The paper is well-written in general and the authors have done a good job of communicating their ideas.***

***Comment: Your abstract does not highlight the specifics of your research or findings. There needs to be an explicit research objective stated, preferably as a separate section.***

**Response:** The Abstract is rearranged and rephrased accordingly. The Motivation, Problem Statement, Objectives, Methods, Results and Conclusion is now explicitly stated as separate sections.

***Comment: The related work section should be extended to present a critical review of existing techniques highlighting their deficiencies. At present, the literature review is presented in a "this did that" format. There is no flow here. This section should be re-written based on the techniques rather than listing the papers. It is better to provide a tabular format summary of the existing approaches along with strengths and weaknesses as well.*
Response:** The Related work section (Section 2, Subsections 2.1, 2.2 & 2.3) is now presented as per the valuable suggestion of respected reviewer. The tabular summary is also provided accordingly (Table 1, 2 & 3). The strength and weakness of relevant existing approaches are highlighted in Section 3, Example 1 and Table 7.

***For the methodology, it was explained clearly, however it should be supported with an example. The algorithm proposed in the paper has no formal proof that will produce the correct score.*
Response:** Example 2 illustrate the methodology of proposed approach. It presents how successfully the clusters can be obtained using the proposed approach.

***Analyses are missing using more state-of-the-art methods. Compare and provide strong evidence about the efficiency of the proposed approach with similar approaches based on fuzzy bipolar soft set and Pythagorean fuzzy bipolar soft set, so far, no such experimentation is provided.***

**Response:** With all due respect, the goal of this research is to explore some significant limitations in existing rough set based categorical clustering techniques only. Therefore, particularly the cases where these techniques are unable to produce quality clusters (independent and insignificant data) are considered. Accordingly, we suggest a better, viable and more comprehensive alternative approach RPA in our research. Therefore, the analyses of only relevant rough set based categorical techniques like MDA, MSA and ITDR is presented in Section 6, Table 11-19. This comparison provides strong evidence about the efficiency of the proposed approach in terms of several evaluation parameters.

The comparison with other approaches like fuzzy bipolar soft set and Pythagorean fuzzy bipolar soft set is valuable point but due to scope limitations we have mention it as future work in Section 9**.**

***Show the robustness checking of the proposed model. How the proposed approach is effective in terms of computational resources like memory and execution time.***

**Response:** The computational complexity of proposed clustering strategy is determined by the number of iterations required and in terms of respond time. It is mentioned in 2^nd^ last paragraph of Section 1 (Introduction). The computational complexity in terms of Big O is illustrated in Definition 5. Table 11, 12, 14 & 15 shows the relevant experimental results.

***The conclusion is not precise. The key findings and its implementation potential (in practice) is missing. Clearly, identify its academic contributions also. Limitations are not mentioned.***

**Response:** The Conclusion (Section 9) is now rearranged to include key findings, contributions and limitations. Other limitations are highlighted in Section 8.

***While revising your submission, please upload your figure files to the Preflight Analysis and Conversion Engine (PACE) digital diagnostic tool,*** [***https://pacev2.apexcovantage.com/***](https://pacev2.apexcovantage.com/)***.***

**Response:** The Figures are now enhanced using PACE. Thanks for anther valuable suggestion.
